# Temporal patterns in multiple stressors shape the vulnerability of overwintering Arctic zooplankton

**DOI:** 10.1002/ece3.11673

**Published:** 2024-06-30

**Authors:** Albini Dania, Mathieu Lutier, Martin P. Heimböck, Jan Heuschele, Janne E. Søreide, Michelle C. Jackson, Khuong V. Dinh

**Affiliations:** ^1^ Department of Biology University of Oxford Oxford UK; ^2^ Section for Aquatic Biology and Toxicology, Department of Biosciences University of Oslo Oslo Norway; ^3^ Institute of Environmental Medicine Karolinska Institutet Stockholm Sweden; ^4^ The University Centre in Svalbard Longyearbyen Norway

**Keywords:** climate change, copepods, interactive stressors, recovery, temporal dynamics

## Abstract

The Arctic polar nights bring extreme environmental conditions characterised by cold and darkness, which challenge the survival of organisms in the Arctic. Additionally, multiple anthropogenic stressors can amplify the pressure on the fragile Arctic ecosystems during this period. Determining how multiple anthropogenic stressors may affect the survival of Arctic life is crucial for ecological risk assessments and management, but this topic is understudied. For the first time, our study investigates the complex interactions of multiple stressors, exploring stressor temporal dynamics and exposure duration on a key Arctic copepod *Calanus glacialis* during the polar nights. We conducted experiments with pulse (intermittent) and press (continuous) exposure scenarios, involving microplastics, pyrene and warming in a fully factorial design. We observed significant effects on copepod survival, with pronounced impacts during later stressor phases. We also detected two‐way interactions between microplastics and pyrene, as well as pyrene and warming, further intensified with the presence of a third stressor. Continuous stressor exposure for 9 days (press‐temporal scenario) led to greater reductions in copepod survival compared to the pulse‐temporal scenario, characterised by two 3‐day stressor exposure phases. Notably, the inclusion of recovery phases, free from stressor exposure, positively influenced copepod survival, highlighting the importance of temporal exposure dynamics. We did not find behaviour to be affected by the different treatments. Our findings underscore the intricate interactions amongst multiple stressors and their temporal patterns in shaping the vulnerability of overwintering Arctic copepods with crucial implications for managing Arctic aquatic ecosystems under the fastest rate of ongoing climate change on earth.

## Graphical abstract

Our study investigates the impact of multiple stressors on Arctic copepods during polar nights, a crucial yet understudied area. By examining pulse and press exposure scenarios of microplastics, pyrene and warming, we found significant reductions in copepod survival, particularly during later stressor phases. Continuous stressor exposure led to greater harm compared to pulse exposure with recovery phases positively influencing copepod survival. These findings highlight the complexity of stressor interactions and temporal dynamics in shaping the vulnerability of Arctic copepods with significant implications for ecosystem management in a rapidly changing climate.

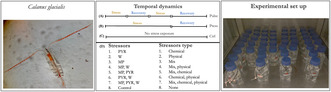

## 1 INTRODUCTION

Ecosystems are increasingly exposed to multiple stressors of varying nature (e.g., chemical pollution and global warming) and temporal patterns (continuous – press vs. sporadic – pulse) due to human activities (Crain et al., 2008; IPCC, 2021; King et al., 2022; Rillig et al., 2021). These stressors have the potential to significantly impact biodiversity, ecosystem functioning and the provision of ecosystem services (Reid et al., 2019; Scherrer et al., 2023). Recent progress has been made in understanding the combined effects of multiple stressors (Albini et al., 2023; Jackson et al., 2016). However, quantifying interactions amongst multiple stressors remains a complex challenge, representing a key problem in ecology and conservation (Dinh et al., 2021; Rhind, 2009). The outcomes of multiple‐stressor interactions can produce complex and unpredictable effects, displaying considerable variability due to contextual factors, such as environmental variability. This has led to inconsistent results in several studies (Dinh et al., 2021; Jackson et al., 2021; Morris et al., 2022), adding further complexity to the understanding and prediction of global change impacts (Lange et al., 2018; Rodrigues‐Filho et al., 2023; Wear et al., 2023). Thus, there is a pressing need for an increased understanding of the interactive effects of multiple stressors to prevent biodiversity loss and ecosystem service degradation.

Stressor identity can play an important role in determining the effects of stressors on an ecosystem (Jackson et al., 2016; Perujo et al., 2021). Stressors can be grouped into four different categories (or identities): (i) physical (e.g., warming, water flow and land use change), (ii) chemical (e.g., nutrients, pesticides and toxic substances), (iii) biological (e.g., invasive species and disease) (Rillig et al., 2021) and (iv) mix of stressors, where overlaps between two stressors categories exist (e.g., microplastics and nanoparticles). Certain combinations of stressors might exhibit interactive effects important to incorporate in future modelling efforts (Halpern & Fujita, 2013; Schäfer et al., 2023). Chemical substances can, for example, interact with physical stressors to amplify their effects. For instance, ingestion of microplastics could increase the transport of contaminants inside aquatic organisms (Wang et al., 2020). Comparing and classifying stressors have the potential to help predict scenarios where multiple stressors produce pronounced interactive effects.

The nature of stressor interactions and the predictability of combined effects can vary based on the presence of ‘time‐lags’ or ‘recovery phases’ between stressor events (Bundschuh et al., 2023; Lau & Hanson, 2023; Vinebrooke et al., 2004). Previous studies have underlined the significance of considering these ‘recovery phases’ in explaining stressor impacts, emphasising the importance of changing temporal dynamics (Ryo et al., 2019). In fact, high temporal variability is common for both natural and human disturbances (e.g., Bundschuh et al., 2023; Jackson et al., 2021). If there is a time gap between the occurrence of one stress event and the next, a system may have a chance to recover between events, resulting in a reduced overall effect compared to simultaneous events. On the other hand, if the system experiences repeated stress events without sufficient recovery time, it may be more severely affected. Although previous experimental studies have focused on cumulative individual stressors (e.g., Bulleri et al., 2014; Hughes et al., 2018), there has been limited exploration and testing of these ideas concerning the temporal regimes of interactive stressors (see Dinh et al., 2016). Additionally, little research has explicitly examined the role of time lags between stressor exposures versus constant prolonged stress exposure. Thus, quantifying the effects of interactions amongst variable temporal patterns and stressor identity on ecosystems facing multiple concurrent stressors represents a critical yet challenging step towards understanding the mechanisms governing multiple‐stressor interactions.

The Arctic aquatic environment is a perfect system to investigate the nature and the complexity of multiple rising interacting stressors, as it is an ecosystem that is undergoing rapid changes due to increased exposure to multiple stressors (Bakke et al., 2013; Botterell et al., 2022; Dinh et al., 2019; Post et al., 2019). These stressors include significant shifts in temperature and sea ice cover due to the inflow of warm Atlantic currents, which have profound effects on ice‐dependent species such as zooplankton copepods (Li et al., 2023; Liu et al., 2019). The melting of sea ice is, in turn, opening the Arctic Ocean to increased development of human activities, such as oil exploitation and marine transportation, introducing a new set of stressors to the region (Gauthier et al., 2021; Huntington et al., 2023). These additional stressors could accelerate the impacts of ongoing climate‐related stressors. One notable consequence of increased marine transportation is the potential for the release of ship‐related chemical pollution and microplastics into the sea (Emberson‐Marl et al., 2023; Zhang et al., 2023). Microplastic pollution is a growing environmental concern, with reports indicating an increasing quantity of litter dispersing into secluded environments, including Polar Regions (Cózar et al., 2017; Zhang et al., 2023). Ships contribute to this problem by releasing microplastics through various means, such as paint, equipment like ropes and greywater, including washing machine effluent. Furthermore, the exploration of oil and gas reserves in the Arctic Ocean has led to the release of chemical pollution (Bakke et al., 2013). Oil‐contaminated water from these activities often contains polycyclic aromatic hydrocarbons (PAHs), including pyrene (Reddy & Quinn, 2001; Sørensen et al., 2023). The negative effects of PAHs on various marine organisms have been previously documented (Albani et al., 2023; Sørensen et al., 2023), with their toxicity varying with temperature, although some studies have found contrasting trends (e.g., Dinh et al., 2019).

Therefore, it is crucial to comprehend the effects of these multiple arrays of stressors on Arctic marine organisms to predict the consequences of climate change accurately. In this context, Arctic *Calanus* spp. copepods play a pivotal role as they dominate the planktonic grazer biomass, are exceptionally adapted to polar regions and are sensitive to various stressors and chemical pollutants (Karnovsky et al., 2010; Kosobokova & Hirche, 2009; Sørensen et al., 2023). They serve as the link between primary production and higher trophic levels and contribute significantly to the carbon export and recycling of nutrients to the deep sea (Jónasdóttir et al., 2015, 2022; Steinberg & Landry, 2017). The exposure to stressors can adversely affect their life cycle and could thus decrease the efficiency of the marine food web. Starting from the copepodite stages III, IV and V, Arctic copepods enter a period of dormancy, termed diapause, during the winter months when food is limited (Falk‐Petersen et al., 2007; Freese et al., 2017; Madsen et al., 2008). During diapause, metabolic and digestive enzyme activities decrease substantially, and they rely solely on their internal lipid reserves for sustenance (Auel et al., 2003; Conover & Siferd, 1993; Freese et al., 2017). In this period of resting stage, if copepods experience elevated temperatures due to the inflow of warm Atlantic water, their metabolism likely increases and lipid stores may deplete more rapidly (Werbrouck, 2016). Furthermore, Arctic *Calanus* copepods can accumulate toxic compounds, such as pyrene, within their large lipid reserves (Wezel & Opperhuizen, 1995). These substances are known to be toxic, carcinogenic, mutagenic, and potentially lethal to invertebrates including copepods (Sørensen et al., 2023; Toxværd et al., 2018, 2019). Laboratory studies have also demonstrated that several zooplankton species readily ingest high concentrations of microplastics, leading to a range of detrimental effects, including reduced feeding behaviour, growth and fecundity (Bermúdez & Swarzenski, 2021; Botterell et al., 2022; Cole et al., 2016). Swimming behavioural changes, such as a reduction in the distance travelled, have also been considered an important response to environmental change (Tuomainen & Candolin, 2011). Monitoring behavioural changes can detect early warning signals about impending threats. In recent years, the importance and complexity of behavioural responses to contaminants and their feedback on individual and population levels has been recognised (Saaristo et al., 2018).

Most research on the Arctic marine organisms has focused on the spring and summer period when *Calanus* spp. feed, grow and reproduce (Falk‐Petersen et al., 2009). Nonetheless, it is crucial to recognise that significant biological processes also occur during the winter months (Berge et al., 2020; Dinh et al., 2023). For instance, during the winter season, non‐consumptive mortality (mortality not linked to predation) of *Calanus* copepods is more pronounced compared to other times of the year due to energy‐demanding investment in moulting and gonad development (Daase et al., 2014; Maud et al., 2018). Furthermore, the winter season is particularly significant in the context of human‐induced climate change and other anthropogenic stressors that can affect marine organisms in unforeseeable ways (Dinh et al., 2023; Studd et al., 2021). In fact, winter can interact and alter the type or the magnitude of multiple stressor interactions, giving rise to intricate new interactions (Dinh et al., 2023; Studd et al., 2021). For instance, In the Arctic marine ecosystem, the interactive effect of ocean warming and acidification on the sea snail *Limacina helicina* was additive in the autumn, but synergistic in winter (Lischka & Riebesell, 2012). Consequently, to the best of our knowledge what occurs after copepods descend to their winter dormancy at depth remains extremely limited.

In this study, we conducted a large, fully factorial laboratory experiment to quantify the interactive effects amongst variable temporal patterns of three of the important and rising stressors on the marine aquatic ecosystem in winter: warming (physical stress), pyrene (chemical stress) and microplastics (mix stress). Specifically, we assessed the influence of these single and combined stressors under three distinct temporal regimes (press and temporally variable pulses and a control treatment without stress) on the winter survival and swimming behaviour of the Arctic copepod *Calanus glacialis* during overwintering. We choose to focus on a population‐level parameter such as survival and behaviour because this parameter governs responses that directly affect community and ecosystem‐level dynamics (Caquet & Lagadic, 2021; Udevitz et al., 2013). Our study addresses the following questions: (1) What are the single and combined effects of pyrene, microplastics and warming on overwintering Arctic copepods? (2) Does the presence of recovery‐ or ‘lag’‐phase after a first stressor event influence organisms' responses to a second stressor event during wintertime? (3) Does the duration of stressors exposure impact responses to single and combined stressors?

## 2 MATERIALS AND METHODS

### 2.1 Study organisms: Arctic copepods

**FIGURE 1:**
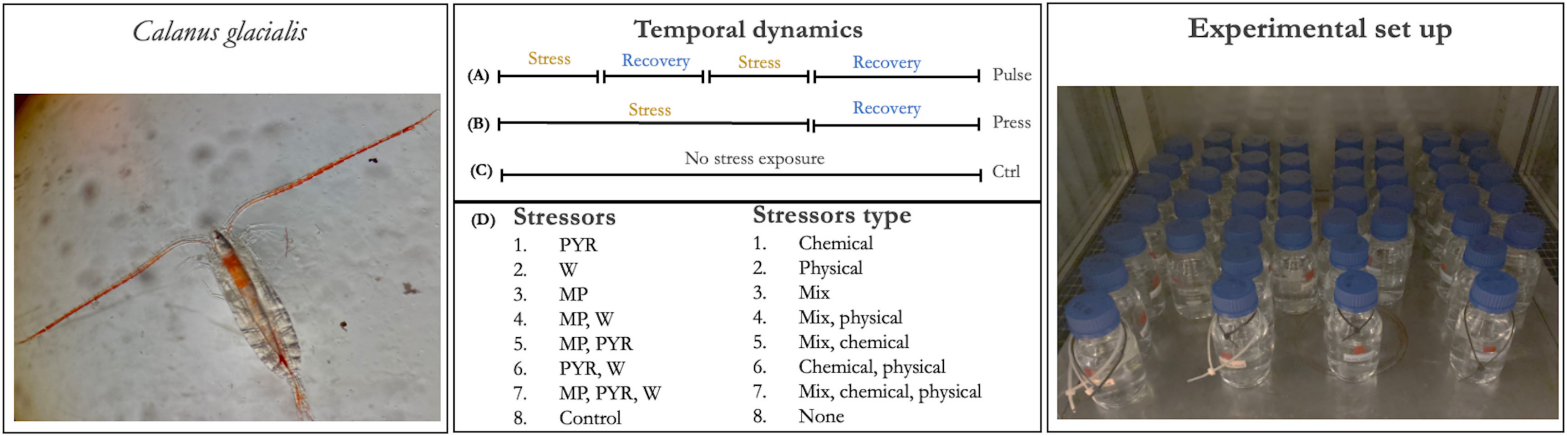
*Calanus glacialis* copepods were subject to an array of different stressors, alone or in combinations: warming (W), microplastics (MP) and pyrene (PYR) in a full factorial design, with three temporal regimes (1): Pulse (2 Pulses & 2 Recovery), Press (continuous exposure with a recovery at the end of the stress application). A control treatment (Ctrl) was set and kept without stressor exposure. The experiment lasted 14 days.

Sampling was conducted on 15th January 2023, near Billefjorden (78°40′N, 16°40′E) in the Arctic, from the RV Helmer Hanssen. Copepods were collected from a depth of 130 m to the surface using a 200 μm net (WP2 Zooplankton Net). Once on the ship, copepods were kept at 3°C and carefully transported to a controlled‐temperature room (3°C, dark) at the University Centre in Svalbard (UNIS) within a day. Once at UNIS, specimens were sorted using stereoscopes and individuals in CIV and CV stages of the marine copepod *C. glacialis* (Figure 1). These copepods were then placed in 2 L bottles filled with filtered seawater. Subsequently, copepods were transported to the University of Oslo (Norway) for the experiment, with temperature control units ensuring the maintenance of a stable temperature of approximately 3°C throughout transport.

### 2.2 Experimental setup (Figure 1)

The experiment took place in February 2023 at the Section for Aquatic Biology and Toxicology, Department of Biosciences, University of Oslo, Norway. We experimentally exposed *C. glacialis* to three stressors: warming, microplastics and pyrene, using a fully crossed design. We conducted the experiment by examining three distinct temporal regimes: pulse, press and a control group with no exposure to stressors.

In the pulse‐temporal treatment, stressors were applied twice, each for a duration of 3 days. There were two recovery phases (i.e., without stressors exposure), included in this treatment. The first recovery phase occurred between the two stressor phases and lasted for 3 days. The second recovery phase took place after the second stressor phase and extended for a longer period than the first one (3 vs. 5 days) to ensure the record of a possible recovery. In total, the pulse‐temporal treatment spanned 14 days (Figure 1a).

The press‐temporal treatment involved continuous exposure to the stressors for a period of 9 days without interruption. Following this continuous‐temporal exposure, there was a single recovery phase that lasted for 5 days. The entire duration of the press‐temporal treatment was also 14 days (Figure 1b).

The specific stressor levels applied in both temporal regimes were as follows: (1) warming (W): applied at 8°C; (2) microplastics (MPs): maintained at a concentration of 200 MPs per millilitre (200 MPs mL^−1^); and (3) pyrene (Pyr): applied at a concentration of 200 nanomolar (200 nM) (Figure 1d).

### 2.3 Treatment preparations: Microplastics (MPs), warming (W) and pyrene (PYR) stressors

Commercial polystyrene spheres measuring 30 μm in size were purchased from Sigma–Aldrich in a liquid solution (84135‐5 mL‐F) and used to create a stock solution of 200 particles per mL^−1^. This size of microplastics is commonly used in cosmetics and personal care products (Fendall et al., 2009), and it has previously been shown to be readily ingested by copepods (e.g., *C. helgolandicus*, Cole et al., 2016). To verify the particle concentration, we used a Benchtop FlowCAM® 8000 (Fluid Imaging Technologies, Inc., Maine, USA). For the microplastic treatment, the stock solution bottle was vigorously shaken and rotated to mix the solution, before adding 25 mL of it to the experimental bottles of 250 mL.

The experiment involved two temperature conditions: 3 ± 0.5°C (control, e.g., Toxværd et al., 2018; Yadetie et al., 2022) and 8 ± 0.5°C (treatment). To create and maintain these temperatures, the experimental bottles were placed in two temperature‐controlled incubators (Sanyo incubator) equipped with a Temperature HOBO Data Logger (UTBI‐001, Onset, Bourne, MA) each. The choice of 8°C for the treatment was based on predictions from the Arctic Climate Impact Assessment (ACIA) (2005) for future Arctic climates and recent predictions (Coupled Model Intercomparison Project phase 6 – CMIP6 models, e.g., Hahn et al., 2021).

The pyrene stock solution at a concentration of 200 mM was prepared by dissolving 20.2 mg of pyrene granulate (purity ≥99%, Sigma–Aldrich, Darmstadt, Germany) in 50 mL of absolute acetone (purity ≥99.8%, VWR International). For comparison, we added acetone (0.05%) to the control treatments without pyrene. This solution was stored at 3°C in darkness to prevent pyrene degradation due to light exposure. A 25 μL of the stock solution was added to the experimental bottles filled with 250 mL FSW, resulting in an exposure solution with a concentration of 200 nM. This concentration was chosen based on previous studies, which showed a reduction of mortality after short‐term exposure (Toxværd et al., 2018), and it can be found in seawater affected by oil exploitations, oil spills or in areas with high shipping activities (Krause et al., 2017).

Initially, we established 12 replicates for each single and combined treatment and for a control group, all maintained in controlled temperature incubators with no light. Each bottle was stocked with 9 copepods and a specific single or combination of stressors assigned to it. The natural seawater used was collected from the Drøbak Aquarium, situated in the inner Oslofjord (Oslo, Norway) and filtered with a 0.22 μm filter (Pentair Industrial BP‐410‐1) and ultraviolet treated (Deltec® Type 391, 39 W) on the same day of collection.

### 2.4 Sampling procedure

Sampling was conducted every 3 days from day 0 to day 9, and the final samples were collected on day 14. To ensure a controlled environment, the laboratory remained in darkness throughout the experiment to mimic natural winter conditions, and sample handling was performed under red‐light conditions (≤8 min bottle^−1^) with an illumination level of approximately 0.3 μmol photons m^−2^ s^−1^ (ULM‐500 Universal Light Meter, Heinz Walz, Effeltrich, Germany).

To assess copepod mortality, the contents of each bottle were gently poured into a plastic box using a 40‐μm nylon mesh. Live copepods were counted and carefully transferred with a pipette to a new bottle containing chilled FSW and their corresponding treatment. Copepods were considered dead if they showed no response to physical stimulation and appeared opaque. After the survival assessment, three bottles were randomly chosen at each sampling point and taken out of the experimental set for the analysis of faecal pellets and behaviour. This process resulted in the initial setup of 12 replicates for each treatment (total of 180 bottles), reducing to 3 replicates per treatment by the end of the experiment (total of 45 bottles). We also checked whether *C. glacialis* were grazing and swimming during the overwintering by checking the faecal pellet production and movement behaviour. For faecal pellet collection, copepods were carefully removed and the bottles were rinsed gently. The faecal material was then preserved in approximately 100 mL of 70% ethanol and checked for the presence versus absence of faecals under the microscope. The copepods left from the bottles were used to examine whether copepods were active during the overwintering and whether the stress treatments may alter their overwintering behaviours, specifically in terms of the distance travelled and the proportion of time spent swimming. Each individual copepod was placed in a cell culture plastic bottle (*n* = 3 per treatment), and we recorded each bottle from the front in darkness for 2 mins using an infrared‐sensitive camera (Raspberry Pi NoIR Camera v2, 25 fps). Illumination was provided by near‐infrared light LEDs, and the field of view included the entire water body. Due to the short length of the process, the water temperature inside the bottle did not differ from the respective treatment temperatures. We extracted the position from each video sequence in the bottle of the copepod using a custom Python script in the Open‐Source Computer Vision Library (OpenCV; version 4.0.0) for each frame, adapted from Wolf and Heuschele (2018), and we calculated the travelled gross distance per individual and the proportion of time spent swimming, from the position data.

### 2.5 Statistical analyses

All statistical analyses were performed using R version 12.0. 353 (R Core Team, 2021). To evaluate how individual and combined stressors affected copepod survival under different temporal dynamics (pulse vs. press) and across various stress and recovery phases, we used a generalised linear model (GLM) analysis with a Poisson distribution. In this analysis, the number of survivors served as the dependent variable, and treatments were considered fixed effects.

We examined how the presence of a recovery influenced the copepods, using the survival data during the final stressor phases (Phase II for the pulse‐temporal scenario and Phase III for the press‐temporal scenario). These phases were chosen because copepods experienced at least one stressor exposure event (one for the press‐temporal scenario and two for the pulse‐temporal scenario). To examine this relationship, we conducted a linear regression analysis, with previous recovery and treatment as fixed factors and the weighted survival of the copepods, accounting for exposure time, as the dependent variable. The weighted survival was calculated by dividing the number of survivors in each replicate by the corresponding exposure time. We conducted the Shapiro–Wilk test and Levene's Test to assess the normality of residuals and the homogeneity of variance, respectively. These tests confirmed that the assumptions of normality and equal variance were satisfied. As we observed a significant impact of recovery, we further conducted a two‐sided *t*‐test to compare how copepods responded to each of the three stressors individually, either with or without prior recovery, using the exposure length‐weighted survival calculated as described above.

To determine if the duration of stressor exposure influenced copepod responses, we initially tested the effects of consecutive exposures (I, II and III) on copepod survival for the press‐temporal dataset. This was done using a GLM analysis with a Poisson distribution, with Exposure ID (I, II or III) and treatments as fixed effects. We did not perform this analysis on the pulse‐temporal dataset because the stressor phases were separated by a recovery period and thus lacked continuous stressor exposure.

To test if copepods are more active when exposed to a single or combination of stressors, we used a GLM analysis with a Gamma (link = ‘log’) distribution, with the stressor treatments and the temporal exposure (control, press and pulse) as fixed effects and the gross distance travelled (mm) and the proportion of time spent swimming as a dependent factor.

Visualisations of results were performed using the package ‘ggplot2’ (version 3.4.2, Wickham, 2016).

## 3 RESULTS

### 3.1 Effect of stressors and temporal dynamics on copepod survival

**FIGURE 2:**
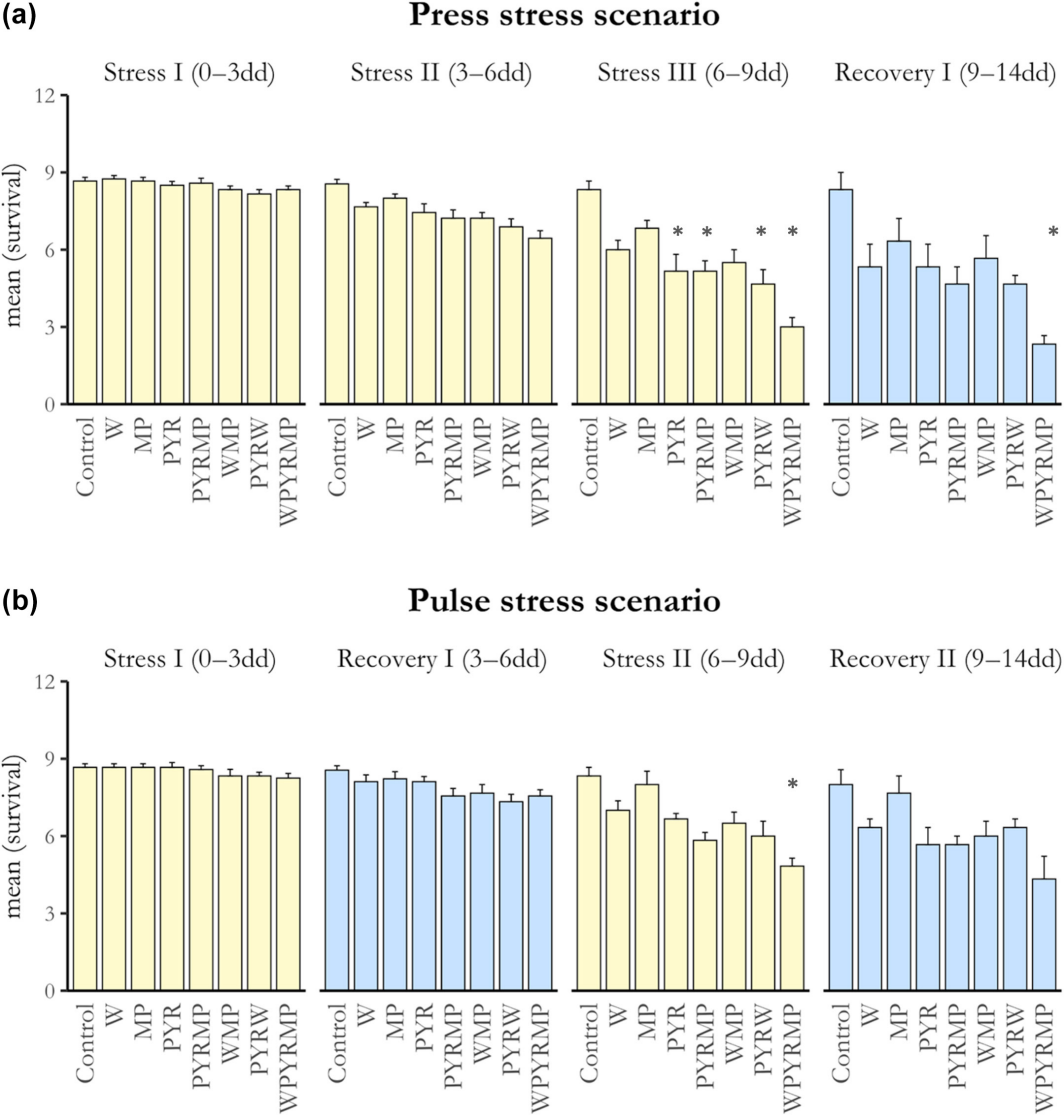
Effects of individual and all the stressor combinations on *C. glacialis* average survival (mean survival, *y*‐axes) starting with 9 copepods per bottle at Day 0. Stressor phases are denoted in yellow, whilst recovery phases are in blue. Panel (a) illustrates the pulse‐temporal stress scenario, whilst panel (b) shows the press‐temporal stress scenario. The length for each phase is reported in brackets, in days (dd). Asterisks (*) indicate the statistical differences between the control and the treatments based on the number of survivals (Table S1).

Overall, copepod survival was significantly more affected by stressor exposure in the press‐temporal scenario compared to the short‐term exposure scenario (pulse‐temporal) when considering all combinations of stressors (Figure 2, Table S1). Notably, the most substantial impact of stressors on copepod survival occurred during the final stressor phases (Phase III for press‐temporal and Phase II for pulse‐temporal exposure). As anticipated, the initial phases of stressor exposure in both temporal stress scenarios (Phase I) had no significant effect on copepod survival (Figure 2a,b).

Within the press‐temporal scenario, we saw an effect of stressors only in the final stressor phase (i.e., Stressor Phase III). Pyrene alone had a significant impact during this latter phase (*z* = −2.091, *p* = .04), whilst microplastics and warming had no significant effects (Table S1). Furthermore, the combined presence of pyrene and microplastics resulted in a significant reduction in copepod survival (*z* = −2.091, *p* = .03), as did the combination of pyrene and warming (*z* = 0.236, *p* = .01), and the presence of all three stressors together (*z* = −3.717, *p* < .01) during the final stressor phase. The stronger reduction of survival happened with the exposure to the three stressors combination in Stressor Phase III (Mean Survival WPYRMP = 3.0 ± 0.3 SE, Mean Survival PYRMP = 5.16 ± 0.40, Mean Survival PYRW = 4.66 ± 0.55; Figure 2b).

In the pulse‐temporal scenario, only the interaction of the three stressors had a significant effect on copepods' survival during the second stressor phase (i.e., Stressor phase II, *z* = −2.334, *p* = .02, Figure 2a). None of the other stressors singularly or in combination influenced the survival of the copepods (Table S1).

During the recovery phase, copepod mortality was low and generally unaffected by prior exposure to stressors, except in the press‐temporal scenario, where the interaction of all three stressors continued to influence copepod survival (*z* = −2.977, *p* = .02, Figure 2b).

We could not detect any consistent effects of the treatments nor the scenarios on the travelled gross distance and on the proportion of time spent swimming for the copepods at any of the sampling days (Table S2).

### 3.2 Effect of a recovery phase on copepod survival

In our study, we observed a significant impact of the presence of a recovery phase, characterised by the absence of stress exposure, on the survival of *C. glacialis* (*F*_1,80_ = 275.99, *p* < .05). However, no interactive effect of previous recovery and treatment was found (*F*_7,80_ = 0.39, *p* = .90). When comparing the survival at the two temporal scenarios (i.e., with and without a recovery phase), we found higher survival for copepods subjected to the recovery phase (i.e., pulse‐temporal) for all stressors assessed (Figure 2a,b). *T‐test* analyses revealed that this pattern was consistent across all three stressors applied singularly. In the case of pyrene exposure, copepods in the pulse‐temporal stressor exposure scenario had an average survival of 6.66 ± 0.21 SE, which was higher than the average survival of 5.16 ± 0.65 SE observed in the press‐temporal scenario (*t* = 6.653, *p* < .01). A similar trend was observed for warming, where copepods in the pulse‐temporal scenario displayed a mean survival of 7.0 ± 0.36 SE compared to the lower average survival of 6.0 ± 0.36 SE in the press‐temporal exposure (*t* = 6.836, *p* = <.05). Similarly, when exposed to microplastics, copepods in the pulse‐temporal scenario showed a higher survival compared with the press‐temporal scenario (8 ± 0.51 SE vs. 6.83 ± 0.30 SE, *t* = 6.2, *p* = <.05). Two‐ and three‐ways interactions saw a similar trend, with the survival being higher in the pulse‐temporal stressor scenario compared with the constant exposure of stressors (PYRMP: *t* = 5.862; *p* = <.05, WMP: *t* = 5.221, *p* = <.05; PYRW: *t* = 4.206, *p* = <.05; WPYRMP: *t* = 7.227; *p* = <.05).

#### 3.2.1 Influence of exposure duration on copepod survival

**FIGURE 3:**
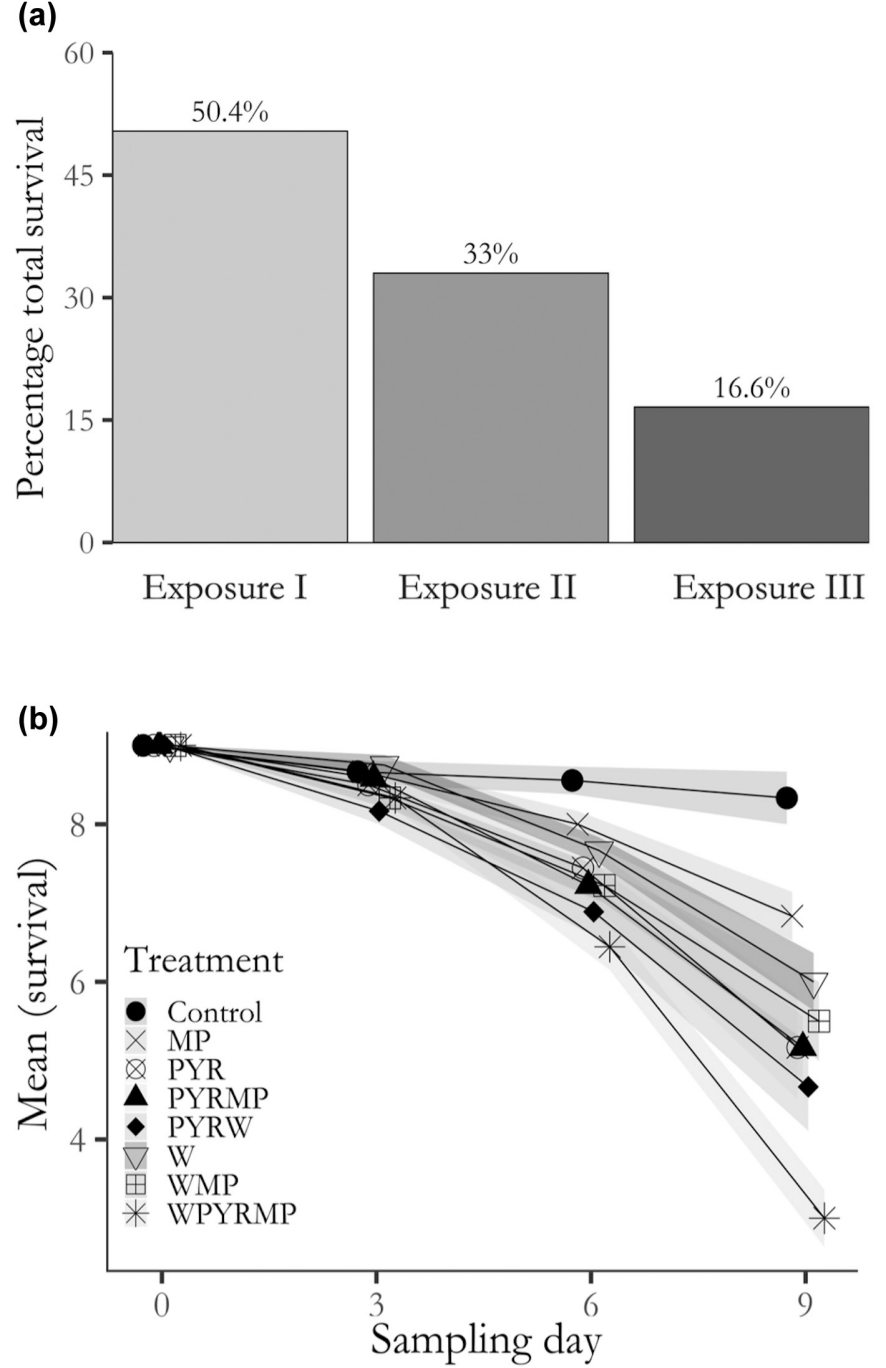
Decrease in *C. glacialis* mean survival in the press‐temporal scenario. Panel (a) shows the cumulative percentage of total survival in the three subsequential exposures. Panel (b) represents the mean copepod survival (mean ± SE) for all the combinations of multiple stressors over the three subsequential stressor exposures. Shapes indicate the different treatments at each sampling point and the grey areas are the 95% confident intervals for each treatment.

Our analysis revealed a significant impact of exposure duration across three stressor phases in the press‐temporal scenario, on the mean survival within each treatment (*χ* ^2^ = 38.13, *p* < .05, Figure 3b), as well as in the cumulative survival value (Figure 3a). Specifically, the mean survival of copepods in the different stressor treatments decreased progressively from stressor phase I to stressor phase III (Figure 3b). The most significant decrease in survival occurred on day 9, driven by a three‐way interaction, followed by the high reduction in survival in the treatments with an interaction of warming and pyrene (Figure 3b, black prism symbol). Copepods in the control treatments maintained the highest survival consistently over the entire 9‐day period (Figure 3b, Table S1). Consequently, the total cumulative copepod survival in the three stressor exposures followed a similar trend, decreasing from stressor phase I (50.4%) to stressor phase III (16.5%), with stressor phase II displaying an intermediate total value of 33.0% (Figure 3a).

## 4 DISCUSSION

The results of our study show the complex interplay between three rising stressors, namely warming, pyrene and microplastics, and the importance of temporal exposure patterns in shaping the responses of overwintering Arctic marine copepods *C. glacialis* (Dinh et al., 2023). These findings contribute to our understanding of how aquatic organisms cope with stressors which are variable in time and kind over the winter period. These results have important implications for the management of Arctic aquatic ecosystems in the face of increasing global warming.

### 4.1 Effect of stressors, temporal dynamics and exposure duration on copepods

Our study investigated the influence of stressors, namely pyrene, warming and microplastics, on the survival of the Arctic copepod *C. glacialis*, during overwintering. Our findings align with prior research (e.g., Cole et al., 2015, 2016), indicating that these stressors have a significant impact on copepod survival, including their individual effects and various two and three‐way interactions. Whilst previous research has demonstrated the adverse effects of warming during the summer on copepod survival (Garzke, 2014), our research focused on the much less‐explored impact of warmer winters on overwintering copepods. Interestingly, we did not observe a decrease in copepod survival when warming was applied in isolation (Table S1). This result may be attributed to copepodite stages' lower sensitivity to warming compared to naupliar stages, thanks to their thicker and less permeable cuticle and a relatively lower surface‐to‐volume ratio (Grenvald et al., 2013; Sverdrup et al., 2001).

However, investigating the potential effects of warming on overwintering copepods across different life stages remains relevant. Elevated temperatures can disrupt copepod metabolism, potentially reducing lipid storage capacity and affecting the duration of winter diapause (Dinh et al., 2021; Pierson et al., 2013; Wilson et al., 2016). Our study revealed that when copepods were exposed to high water temperatures in combination with pyrene, either alone or in a three‐way combination with microplastics, their survival decreased. This higher mortality can be explained by the fact that temperature can enhance the toxicity of pollutants (Noyes et al., 2009). Whilst synergistic effects of marine warming and pyrene have been documented in other copepod species (e.g., *Centropages velificatus*, Hernández‐Avila et al., 2021), our study is the first to report their combined effects on overwintering Arctic copepods.

Notably, research on the effects of pyrene on Arctic copepods has focused on the reproductive season, highlighting detrimental impacts such as reduced survival and lipid mobilisation (Sørensen et al., 2023; Wezel & Opperhuizen, 1995). Our experiment uncovered a significant decrease in the survival of overwintering copepods when exposed to pyrene in wintertime. The toxicity of pyrene involves its interaction with cellular mechanisms, leading to long‐term damage, immobilisation, reduced survival of nauplii and adults and decreased ingestion and reproduction rates (Rist et al., 2024; Toxværd et al., 2018). Moreover, we demonstrated that pyrene and other PAHs may interact with other stressors, consistent with prior research (e.g., Cole et al., 2015; Dinh et al., 2019; Rist et al., 2024). The growing concern regarding microplastic pollution and its potential to increase the bioavailability and transport of organic contaminants in aquatic environments (Batel et al., 2020; Wang et al., 2020) prompted our exploration of the interaction between pyrene and microplastics (Guven et al., 2018). Further, Wang et al. (2020) reported that pyrene and microplastics exhibit a high level of compatibility, with pollutants adhering to their surfaces and organisms mistakenly ingesting them as potential food sources. In our study, we observed a significant interaction between these two stressors, corroborating the notion that copepods may ingest microplastics, especially during overwintering when food availability is limited. Furthermore, we observed that copepods exposed to both stressors (i.e., microplastics and pyrene) started to produce faecal pellets. Notably, microscopic analysis revealed that a substantial portion of these pellets contained microplastics (Figure S1, unpublished data). Unsurprisingly, the survival of copepods was not significantly influenced by microplastics alone. This outcome might be attributed to the relatively lower concentration of microplastics used or the length of exposure time chosen in our study compared to previous research (e.g., Cole et al., 2016). Our choice of a lower concentration aimed to examine the fate and effects of microscopic plastic under controlled laboratory conditions without reaching extreme lethal levels seen in prior ecotoxicological studies (Watts et al., 2014).

The increasing array of human‐induced stressors affecting our ecosystems underscores the need to determine which stressors may have the most severe impacts (Morris et al., 2022). This is crucial for predicting the combined effects of these stressors (Orr et al., 2022). In our experiment, we found that stressors with different origins (e.g., pyrene – chemical and warming – physical) could have a more detrimental effect on copepods survival than stressors that are more similar (e.g., microplastics – physical and chemical and warming – physical). This highlights how interactions between different stressors can potentially have a more detrimental impact compared to stressor combinations that may share some similarities (Morris et al., 2022; Orr et al., 2022). This would imply that organisms, and more specifically copepods, subject to two different stressors would need to put in place stronger defensive strategies to adapt and survive those stressors, compared with facing two similar stressors.

Furthermore, we observed that copepod survival was more affected by the press stressors exposure compared to the pulse‐temporal scenario (Figure 2, Table S1). Only the single exposure and the interaction of warming with microplastics did not affect *C. glacialis* survival by day 9, whilst the other stressor combinations did. This difference may be due to the shorter duration of the two stressor phases in the pulse‐temporal scenario (3 days) compared to the 9‐day exposure in the press‐temporal scenario. Nevertheless, it is essential to investigate the potential effects of short‐term extreme stressor episodes, as they are becoming more and more common due to climate change and more variable weather systems, especially during the less‐studied wintertime (Dinh et al., 2023; IPCC, 2022). Further experiments could explore a more prolonged exposure time than the 3 days used in our experiment for the stressor phases in the pulse‐temporal scenario and a longer recovery phase at the end of the experiment, to test if a longer exposure might induce any influence on the copepod survival and/or delayed effects, especially on later seasons such as the spring growth, development and reproduction.

Despite the strong effects of stressors on the copepod survival, we observed no significant changes in behaviour, specifically in terms of the distance covered and the time spent swimming, across different treatments. Copepods, in general, exhibited low motility during overwintering (e.g., Coguiec et al., 2023). Hirche (1987) documented reduced activity in three Arctic *Calanus* species when exposed to a temperature range between 5 and 10°C. Previous studies have found the negative effect of pyrene on the behaviour of copepods due to the narcotic effects (Grenvald et al., 2013; Jensen et al., 2008), resulting in reduced grazing and ultimately affecting the survival (Grenvald et al., 2013). However, this may not be the case for overwintering *Calanus* as the inherently low activity of overwintering *Calanus* was likely determined by the circadian clock genes (e.g., Hüppe et al., 2020). The insensitivity of overwintering *Calanus* behaviours to stressors may result in increased physiological damages that could explain the observed effect on survival.

### 4.2 Effect of a recovery phase on copepod survival

A key finding from our study revealed that time‐lags between stressors had a significant impact on copepod survival. These temporal gaps had a pronounced effect, amplifying the impact of stressors (reduced survival) when compared to previous exposures in the same pulse‐temporal stress regime. However, these time‐lags reduced the adverse effects significantly when contrasted with continuous (press‐temporal) exposure, resulting in higher survival rates in the pulse‐temporal stress scenario (Figure 2a,b).

In the press‐temporal stress scenario, survival decreased progressively from stressor Phase I to Phase III (Figure 3), underscoring the exacerbation of negative effects on copepod survival during prolonged stressor exposure. In contrast, the presence of a time‐lag, or lag‐phase, between stressors seemed to enable copepods to better cope with subsequent stressor exposures, aligning with previous research (Bertocci et al., 2017; Jackson et al., 2021). The influence of stressor regimes and their temporal patterns on ecological systems has long been recognised as a crucial factor in determining the impact of stressors (Bertocci et al., 2017; Jackson et al., 2021; Orr et al., 2022). Our research highlights the significance of these temporal dynamics between stressors. Notably, we extended our findings to demonstrate that time‐lags between stressors can reduce their impacts, a phenomenon rarely demonstrated before (Brooks & Crowe, 2019). This phenomenon can be attributed to the fact that temporal gaps in stressor regimes offer a pathway for the development of stress‐hardening strategies. These strategies allow organisms to evolve mechanisms to better cope with subsequent stressor events (Kültz, 2015; Vinebrooke et al., 2004). For instance, studies on plants by Walter et al. (2011) demonstrated that plants subjected to an initial short‐term stressor developed a ‘memory’ of the previous stress, resulting in a more rapid response when exposed to subsequent stressors (Jackson et al., 2021; Walter et al., 2011). In our study, copepods in the pulse‐temporal scenario might have set up short‐term acclimation strategies, as indicated by their lower mortality compared to constant stressor exposure. However, it is also possible that the duration of the time‐lag between stress events was insufficient for the full development of hardening (Godbold & Solan, 2013). This would explain the lower average survival during the second stressor phase of the pulse‐temporal stressor scenario (Figure 2) and the enduring effects of the three stressors once removed in the last recovery phase.

## 5 CONCLUSIONS

In conclusion, our study addresses a significant knowledge gap by extending the focus of previous research on multiple stressors from the reproductive season to the often‐overlooked overwintering period, an area that has only recently started to receive attention (see Dinh et al., 2023). Furthermore, our unique experimental design, comparing pulse versus constant temporal stressor/s exposure and exploring the role of lag‐phases in overwintering organisms, provides valuable insights. This novel approach is particularly crucial due to the increasing unpredictability in temporal climate patterns, characterised by heightened variability in extreme climatic events (Easterling et al., 2000; IPCC, 2022).

As climate changes continue to reshape the temporal patterns of stressors in complex ways, it becomes imperative to accurately assess and manage environmental impacts associated with the growing array of human‐induced stressors (Jackson et al., 2021; Schlaepfer & Lawler, 2023). Our study underscores the intricacies of stressor interactions in aquatic ecosystems during the less‐studied yet equally significant winter season on ecological relevant organisms. Monitoring alterations in marine plankton communities can, in fact, help improve our understanding of how rapid changes in plankton might alter marine ecosystem functioning and impact the regulating, provisioning and cultural services ecosystems provide to humans (Botterell et al., 2022; Louchart et al., 2023). For example, disruptions in plankton populations during the overwintering period can affect fisheries and ocean carbon sequestration. To effectively address these challenges, we must adopt management strategies that account for exposure duration, temporal stressor patterns, and the specific nature of stressors. Such an approach will be fundamental in predicting and mitigating the combined effects of stressors on aquatic ecosystems throughout all seasons. As we put our efforts into managing and protecting these ecosystems in the face of global environmental changes, a thorough understanding of these factors is crucial for making informed decisions and conservation efforts.

## AUTHOR CONTRIBUTIONS

**Albini Dania:** Conceptualization (lead); formal analysis (lead); funding acquisition (equal); investigation (equal); methodology (lead); visualization (lead); writing – original draft (lead); writing – review and editing (equal). **Mathieu Lutier:** Investigation (equal); methodology (equal); writing – review and editing (equal). **Martin P. Heimböck:** Investigation (equal); writing – review and editing (equal). **Jan Heuschele:** Conceptualization (equal); methodology (equal); writing – review and editing (equal). **Janne E. Søreide:** Methodology (equal); writing – review and editing (equal). **Michelle C. Jackson:** Conceptualization (equal); funding acquisition (equal); methodology (equal); writing – review and editing (equal). **Khuong V. Dinh:** Conceptualization (equal); funding acquisition (equal); investigation (equal); methodology (equal); resources (lead); writing – review and editing (equal).

## FUNDING INFORMATION

K.V.D. received grants from The Nansen Legacy (RCN #276730) and Researcher Project for Young Talents (RCN #325334) of the Research Council of Norway. D.A. received a Fellowship for Research Stays in Norway for Visiting Researchers (RCN #341499).

## CONFLICT OF INTEREST STATEMENT

The authors declare no competing financial interests.

## Supporting information


Appendix S1

## Data Availability

The data analysed during this study are publicly available in the electronic supplementary material and by request to the corresponding author.
