# Supplementary material for: Temporal patterns in multiple stressors shape the vulnerability of overwintering Arctic zooplankton
Source: Ecol Evol. 2024 Jun 30;14(7):e11673. doi: 10.1002/ece3.11673 (PMC11215157; doi:10.1002/ece3.11673)
Supplement: Supplementary file 1 — Appendix S1 [file ECE3-14-e11673-s001.docx]

**Temporal patterns in multiple stressors shape the vulnerability of overwintering Arctic zooplankton**

Albini Dania*^1,2^, Mathieu Lutier^2^, Martin P. Heimböck^2,3^, Jan Heuschele^2^, Janne E. Søreide^4^, Michelle C. Jackson^1^, Khuong V. Dinh^2^

^1^Department of Biology, University of Oxford, Oxford, OX1 3SZ, United Kingdom

^2^Section for Aquatic Biology and Toxicology, Department of Biosciences, University of Oslo, Blindernveien. 31, 0371 Oslo, Norway

^3^Institute of Environmental Medicine, Karolinska Institutet, 171 77 Stockholm, Sweden

^4^ The University Centre in Svalbard, 156, N-9171 Longyearbyen, Norway

***Correspondence:** Albini Dania, dania.albini@biology.ox.ac.uk

**Supplementary material:**

**Figure S1.** Two copepods faecal pellets collected in the MP treatments during stressors phases. The first one was collected in a replicate of MP during stressor phase II in the press temporal scenario, and the second one was collected during the stressor phase I of the pulse temporal scenario in one of the MP+PYR treatments. Arrows indicate the microplastics.


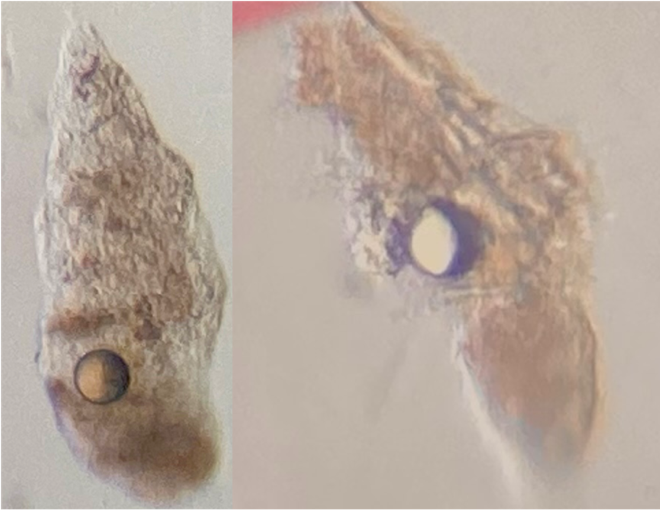


**Table S1.** Results of the GLM analysis to test how singular and combined stressors affected copepod survival in each stress and recovery phases for the two temporal scenario (pulse *vs.* press). MP = microplastics, PYR= pyrene, W= warming.

| ***Press temporal scenario*** | |  |  |
| --- | --- | --- | --- |
| **Stress type** | **Standard Error** | **Z value** | **P value** |
| **D3 – stress phase** |  |  |  |
| Intercept | 9.806e-02 | 22.023 | <2e-16 |
| MP | 9.806e-02 | 0.000 | 1.000 |
| PYR | 1.387e-01 | -0.139 | 0.889 |
| PYRMP | 1.390e-01 | -0.070 | 0.945 |
| W | 1.383e-01 | 0.069 | 0.945 |
| WMP | 1.401e-01 | -0.280 | 0.779 |
| WPYR | 1.408e-01 | -0.422 | 0.673 |
| WPYRMP | 1.401e-01 | -0.280 | 0.779 |
| **D6 – stress phase** |  |  |  |
| Intercept | 0.11396 | 18.836 | <2e-16 |
| MP | 0.16394 | -0.410 | 0.682 |
| PYR | 0.16707 | -0.833 | 0.405 |
| PYRMP | 0.16844 | -1.006 | 0.315 |
| W | 0.16577 | -0.662 | 0.508 |
| WMP | 0.16844 | -1.006 | 0.315 |
| WPYR | 0.17063 | -1.270 | 0.204 |
| WPYRMP | 0.17386 | -1.630 | 0.103 |
| **D9 – stress phase** | | | |
| Intercept | 0.1414 | 14.993 | < 2e-16 |
| MP | 0.2107 | -0.942 | 0.346239 |
| PYR | 0.2286 | -2.091 | 0.036515 |
| PYRMP | 0.2287 | -2.091 | 0.036515 |
| W | 0.2186 | -1.503 | 0.132867 |
| WMP | 0.2243 | -1.853 | 0.063935 |
| WPYR | 0.2360 | -2.456 | 0.014032 |
| WPYRMP | 0.2749 | -3.717 | 0.000202 |
| **D15 – recovery phase** | | | |
| Intercept | 0.2000 | 10.601 | < 2e-16 |
| MP | 0.3044 | -0.902 | 0.36722 |
| PYR | 0.3202 | -1.394 | 0.16333 |
| PYRMP | 0.3338 | -1.737 | 0.08239 |
| W | 0.3202 | -1.394 | 0.16333 |
| WMP | 0.3144 | -1.227 | 0.21989 |
| WPYR | 0.3338 | -1.737 | 0.08239 |
| WPYRMP | 0.4276 | -2.977 | 0.00291 |
|  |  |  |  |
| ***Pulse temporal scenario*** | |  |  |
| **Stress type** | **Standard Error** | **Z value** | **P value** |
| **D3 – stress phase** |  |  |  |
| Intercept | 9.806e-02 | 22.023 | <2e-16 |
| MP | 1.387e-01 | 0.000 | 1.000 |
| PYR | 1.387e-01 | 0.000 | 1.000 |
| PYRMP | 1.390e-01 | -0.070 | 0.945 |
| W | 1.387e-01 | 0.000 | 1.000 |
| WMP | 1.401e-01 | -0.280 | 0.779 |
| WPYR | 1.401e-01 | -0.280 | 0.779 |
| WPYRMP | 1.401e-01 | -0.351 | 0.726 |
| **D6 – recovery phase** | | | |
| Intercept | 0.11396 | 18.836 | <2e-16 |
| MP | 0.16279 | -0.244 | 0.807 |
| PYR | 0.16336 | -0.327 | 0.744 |
| PYRMP | 0.16641 | -0.747 | 0.455 |
| W | 0.16336 | -0.327 | 0.744 |
| WMP | 0.16577 | -0.662 | 0.508 |
| WPYR | 0.16775 | -0.919 | 0.358 |
| WPYRMP | 0.16641 | -0.747 | 0.455 |
| **D9 – stress phase** | | | |
| Intercept | 0.14142 | 14.993 | <2e-16 |
| MP | 0.20207 | -0.202 | 0.8399 |
| PYR | 0.21213 | -1.052 | 0.2928 |
| PYRMP | 0.22039 | -1.618 | 0.1056 |
| W | 0.20931 | -0.833 | 0.4048 |
| WMP | 0.21364 | -1.163 | 0.2448 |
| WPYR | 0.21858 | -1.503 | 0.1329 |
| WPYRMP | 0.23342 | -2.334 | 0.0196 |
| **D15 – recovery phase** | | | |
| Intercept | 0.20412 | 10.187 | <2e-16 |
| MP | 0.29180 | -0.146 | 0.884 |
| PYR | 0.31700 | -1.088 | 0.277 |
| PYRMP | 0.31700 | -1.088 | 0.277 |
| W | 0.30708 | -0.761 | 0.447 |
| WMP | 0.31180 | -0.923 | 0.356 |
| WPYR | 0.30708 | -0.761 | 0.447 |
| WPYRMP | 0.34437 | -1.780 | 0.075 |

**Table S2.** Results of the GLM analysis to test how singular and combined stressors affected copepod behaviour (gross distance travelled and proportion of time spent swimming) in each stress and recovery phases for the two temporal scenario (pulse *vs.* press). MP = microplastics, PYR= pyrene, W= warming, Temporal exposure = pulse *vs* press stress exposure.

| **Gross distance travelled (mm)** | |  |  |
| --- | --- | --- | --- |
| **Stress type** | **Standard Error** | **t value** | **P value** |
| **D3** | | | |
| Intercept | 0.28846 | 17.935 | <0.001 |
| W | 0.36191 | 1.794 | 0.0815 |
| PYR | 0.36191 | 0.265 | 0.7924 |
| MP | 0.37024 | 1.209 | 0.2348 |
| Temporal exposure | 0.15710 | -0.632 | 0.5316 |
| WPYR | 0.46280 | -1.409 | 0.1676 |
| WMP | 0.46158 | -1.400 | 0.1703 |
| PYRMP | 0.46935 | -0.580 | 0.5655 |
| WPYRMP | 0.62423 | 0.468 | 0.6423 |
| **D6** | | | |
| Intercept | 0.25520 | 19.039 | <0.001 |
| W | 0.43246 | 0.328 | 0.745 |
| PYR | 0.36742 | 0.766 | 0.449 |
| MP | 0.35896 | 1.401 | 0.170 |
| Temporal exposure | 0.20746 | 0.279 | 0.782 |
| WPYR | 0.56496 | 0.188 | 0.852 |
| WMP | 0.57977 | 0.576 | 0.568 |
| PYRMP | 0.52001 | 0.306 | 0.762 |
| WPYRMP | 0.78599 | 0.388 | 0.701 |
| **D9** | | | |
| Intercept | 0.36166 | 14.932 | <0.001 |
| W | 0.44841 | -0.143 | 0.887 |
| PYR | 0.45937 | -0.616 | 0.542 |
| MP | 0.45506 | -0.580 | 0.565 |
| Temporal exposure | 0.20864 | -0.785 | 0.438 |
| WPYR | 0.57788 | 0.783 | 0.439 |
| WMP | 0.56531 | 0.656 | 0.516 |
| PYRMP | 0.58465 | 1.104 | 0.277 |
| WPYRMP | 0.82112 | -0.445 | 0.659 |
| **D15** | | | |
| Intercept | 0.28617 | 19.206 | <0.001 |
| W | 0.36049 | -0.793 | 0.433 |
| PYR | 0.36049 | 0.137 | 0.892 |
| MP | 0.36049 | 0.895 | 0.377 |
| Temporal exposure | 0.16869 | -1.029 | 0.311 |
| WPYR | 0.48199 | -0.977 | 0.335 |
| WMP | 0.45334 | 0.465 | 0.645 |
| PYRMP | 0.46266 | -0.191 | 0.849 |
| WPYRMP | 0.63062 | 0.219 | 0.828 |
| **Proportion of time spent swimming** | |  |  |
| **Stress type** | **Standard Error** | **t value** | **P value** |
| **D3** | | | |
| Intercept | 0.22592 | -2.325 | 0.026 |
| W | 0.28345 | 0.751 | 0.457 |
| PYR | 0.28345 | -1.002 | 0.323 |
| MP | 0.28998 | 0.731 | 0.470 |
| Temporal exposure | 0.12304 | -0.466 | 0.644 |
| WPYR | 0.36247 | 0.779 | 0.441 |
| WMP | 0.36152 | -0.321 | 0.750 |
| PYRMP | 0.36760 | 0.395 | 0.695 |
| WPYRMP | 0.48891 | -0.791 | 0.435 |
| **D6** | | | |
| Intercept | 0.21716 | -3.393 | <0.001 |
| W | 0.36800 | -0.497 | 0.62230 |
| PYR | 0.31266 | 0.554 | 0.58292 |
| MP | 0.30546 | 0.222 | 0.82548 |
| Temporal exposure | 0.17654 | 1.145 | 0.25989 |
| WPYR | 0.48076 | 0.648 | 0.52143 |
| WMP | 0.49336 | 0.947 | 0.35017 |
| PYRMP | 0.44251 | 0.107 | 0.91537 |
| WPYRMP | 0.66884 | -0.822 | 0.41644 |
| **D9** | | | |
| Intercept | 0.30896 | -1.691 | 0.0994 |
| W | 0.38307 | -0.067 | 0.9467 |
| PYR | 0.39243 | -0.138 | 0.8906 |
| MP | 0.38875 | 0.162 | 0.8720 |
| Temporal exposure | 0.17824 | -0.237 | 0.8141 |
| WPYR | 0.49367 | 0.513 | 0.611 |
| WMP | 0.48293 | 0.109 | 0.9136 |
| PYRMP | 0.49946 | 0.347 | 0.7305 |
| WPYRMP | 0.70147 | -0.681 | 0.5001 |
| **D15** | | | |
| Intercept | 0.165587 | -2.357 | 0.0245 |
| W | 0.208592 | -1.252 | 0.2192 |
| PYR | 0.208592 | 0.429 | 0.6705 |
| MP | 0.208592 | 1.043 | 0.3047 |
| Temporal exposure | 0.097611 | -1.064 | 0.2952 |
| WPYR | 0.278898 | -0.102 | 0.9194 |
| WMP | 0.262321 | 0.569 | 0.5730 |
| PYRMP | 0.267714 | -0.013 | 0.9900 |
| WPYRMP | 0.364899 | -0.097 | 0.9230 |
